# Meta-Analysis of the Accuracy of Abbreviated Magnetic Resonance Imaging for Hepatocellular Carcinoma Surveillance: Non-Contrast versus Hepatobiliary Phase-Abbreviated Magnetic Resonance Imaging

**DOI:** 10.3390/cancers13122975

**Published:** 2021-06-14

**Authors:** Dong Hwan Kim, Sang Hyun Choi, Ju Hyun Shim, So Yeon Kim, Seung Soo Lee, Jae Ho Byun, Joon-Il Choi

**Affiliations:** 1 Department of Radiology, Seoul St. Mary’s Hospital, College of Medicine, The Catholic University of Korea, Seoul 06591, Korea; kimdh@catholic.ac.kr (D.H.K.); dumky@catholic.ac.kr (J.-I.C.); 2 Asan Medical Center, Department of Radiology and Research Institute of Radiology, University of Ulsan College of Medicine, Seoul 05505, Korea; sykimrad@amc.seoul.kr (S.Y.K.); seungsoolee@amc.seoul.kr (S.S.L.); jhbyun@amc.seoul.kr (J.H.B.); 3 Asan Medical Center, Department of Gastroenterology, University of Ulsan College of Medicine, Seoul 05505, Korea; s5854@amc.seoul.kr

**Keywords:** liver, hepatocellular carcinoma, surveillance, MRI, sensitivity, specificity, systematic review

## Abstract

**Simple Summary:**

Ultrasonography is recommended as a standard surveillance modality, but the performance of surveillance ultrasound for detecting early-stage hepatocellular carcinoma (HCC) is limited. Motivated to provide a more sensitive method, abbreviated magnetic resonance imaging (AMRI) protocols have been introduced for HCC surveillance. We aimed to systematically determine the diagnostic performance of surveillance AMRI for detecting HCC. This meta-analysis of 10 studies comprising 1547 patients found that the pooled sensitivity and specificity of surveillance AMRI for detecting HCC were 86% and 96%, respectively. Hepatobiliary phase contrast-enhanced AMRI showed significantly higher sensitivities for detecting HCC than non-contrast AMRI (87% vs. 82%), but significantly lower specificities (93% vs. 98%). Therefore, surveillance AMRI had overall good diagnostic performance for detecting HCC and might be clinically useful for HCC surveillance. In addition, AMRI protocol should be selected with consideration of the advantages and disadvantages of each protocol.

**Abstract:**

We aimed to determine the performance of surveillance abbreviated magnetic resonance imaging (AMRI) for detecting hepatocellular carcinoma (HCC), and to compare the performance of surveillance AMRI according to different protocols. Original research studies reporting the performance of surveillance AMRI for the detection of HCC were identified in MEDLINE, EMBASE, and Cochrane databases. The pooled sensitivity and specificity of surveillance AMRI were calculated using a hierarchical model. The pooled sensitivity and specificity of contrast-enhanced hepatobiliary phase (HBP)-AMRI and non-contrast (NC)-AMRI were calculated and compared using bivariate meta-regression. Ten studies, including 1547 patients, reported the accuracy of surveillance AMRI. The pooled sensitivity and specificity of surveillance AMRI for detecting any-stage HCC were 86% (95% confidence interval (CI), 80–90%; *I*^2^ = 0%) and 96% (95% CI, 93–98%; *I*^2^ = 80.5%), respectively. HBP-AMRI showed a significantly higher sensitivity for detecting HCC than NC-AMRI (87% vs. 82%), but significantly lower specificity (93% vs. 98%) (*p* = 0.03). Study quality and MRI magnet field strength were factors significantly associated with study heterogeneity (*p* ≤ 0.01). In conclusion, surveillance AMRI showed good overall diagnostic performance for detecting HCC. HBP-AMRI had significantly higher sensitivity for detecting HCC than NC-AMRI, but lower specificity.

## 1. Introduction

Hepatocellular carcinoma (HCC) is the third most leading cause of cancer-related deaths [1], and the incidence of HCC in North America and Europe has risen rapidly over the last 2 decades [2]. Although the prognosis for patients with HCC is quite poor, with an overall 5-year survival rate below 20%, those detected at an early stage are eligible for curative treatments and may have improved survival [3,4]. Therefore, regular surveillance to detect early-stage HCC is generally recommended for at-risk populations [5,6].

Updated guidelines recommend ultrasonography (US) as a standard tool for HCC surveillance [5,6,7]. However, the sensitivity of US for detecting early-stage HCC is not high (47%) [8]. Given this limitation of US surveillance, the recent guidelines suggest alternative surveillance tools, including magnetic resonance imaging (MRI), in selected patients with a high probability of having an inadequate US examination [5,6].

Recent studies showed that surveillance MRI had a higher sensitivity than US for detecting early-stage HCC [9], and it might be more cost-effective than US in patients with virus-associated compensated cirrhosis with a sufficiently high risk of HCC [10]. However, due to its cost, the long exam time, and complexity, the broad application of complete MRI with full sequences is likely to remain limited in a surveillance setting. In this context, abbreviated MRI (AMRI) protocols using a small number of selected sequences that can reduce scanner time and present a lower cost have been introduced [11,12,13].

AMRI protocols can be divided into two categories according to the image sequences included, the first being contrast-enhanced hepatobiliary phase (HBP)-AMRI and the second being non-contrast (NC)-AMRI. HBP-AMRI is conducted after administration of a hepatobiliary agent, i.e., gadoxetate disodium, and consists of T2-weighted imaging (T2WI) and HBP imaging with or without diffusion-weighed imaging (DWI). NC-AMRI consists of up to three sequences from DWI, T2WI, and T1-weighted dual gradient-echo imaging, without the use of contrast media. Given the increased attention to AMRI in HCC surveillance, it is time to clearly determine the performance of AMRI, especially according to the type of protocol. Although a recent meta-analysis reported comparable performance between the two AMRI protocols [14], this result is limited in application to clinical practice for HCC surveillance as it not only includes studies conducted in surveillance patient cohorts, but also studies conducted in diagnostic cohorts that simulate the surveillance setting. Therefore, our study aimed to determine the performance of surveillance AMRI for detecting HCC, and to compare the performance according to different protocols.

## 2. Materials and Methods

This study followed the Preferred Reporting Items for Systematic Reviews and Meta-Analyses (PRISMA) guideline for conduct and reporting [15]. The following literature search, study selection, data extraction, and study quality assessment were independently conducted by two reviewers (both with ≥3 years of experience in meta-analysis and ≥9 years of experience in liver MRI), with all discrepancies being resolved by consensus.

### 2.1. Literature Search Strategy

Thorough searches of MEDLINE, EMBASE, and Cochrane databases were conducted to find studies investigating the diagnostic performance of surveillance MRI using an abbreviated protocol for the detection of HCC. The search query was developed to provide a sensitive literature search. In order to narrow down the number of relevant articles, the identified articles were manually evaluated. The search terms included “Hepatocellular carcinoma”, “MRI”, “abbreviate”, “Surveillance”, and “Screen” (Table S1). The beginning date for the literature search was 1 January 2000, and the search was updated until 3 December 2020. The search was limited to original studies on human subjects written in English.

### 2.2. Eligible Criteria

After removing duplicates, the articles were reviewed for eligibility according to the following criteria: (1) population: patients at risk of HCC without prior history of HCC; (2) index test: liver MRI with abbreviated protocols; (3) reference standard: clinical diagnosis or pathological diagnosis; and (4) outcomes: diagnostic accuracy, including both sensitivity and specificity of AMRI for detecting HCC. Patients at risk for HCC included patients with cirrhosis or chronic liver disease [5,6]. Surveillance was defined as the repeated use of the index test with a regular time interval for the detection of previously undiagnosed lesions [8], and studies performing evaluations for diagnostic purposes instead of surveillance were excluded in our study. The exclusion criteria were as follows: (1) review articles, case reports, protocols, editorials, or conference abstracts; (2) studies that were not within the field of interest; (3) studies not reporting sufficient information to make a diagnostic 2 × 2 table of the imaging results and reference standard findings; and (4) studies with overlapping patient cohorts and data. Articles were first screened by titles and abstracts, and fully reviewed after the first screening.

### 2.3. Data Extraction

The following data were extracted: (1) study characteristics (authors, published year, study country, and study design (retrospective vs. prospective)); (2) subject characteristics, including sample size, age, sex, underlying liver disease, prevalence of HCC, and lesion size; (3) MRI techniques, including MRI sequences, scanner field strength, and interpretation method of AMRI (simulation vs. clinical practice); (4) details of reference standards; (5) surveillance strategies, including repeated surveillance, surveillance interval, and follow-up time; and (6) outcomes, i.e., the accuracy of AMRI for detecting HCC. To determine diagnostic accuracy, the numbers of true-positive, false-positive, true-negative, and false-negative hepatic lesions were counted. When these were not explicitly reported, data were manually extracted using the text, tables, and figures.

### 2.4. Evaluation of Study Quality

The quality of the included articles was evaluated using the Quality Assessment of Diagnostic Accuracy Studies (QUADAS-2) tool [16]. The QUADAS-2 tool assesses study quality according to the four different domains (patient selection, index test, reference standard, and flow and timing). Studies with a high risk of bias in any domain were considered to have a high overall risk of bias.

### 2.5. Summary estimates synthesis

To determine the performance of AMRI for detecting any-stage or early-stage HCC, the sensitivity and specificity with 95% confidence intervals (CIs) were calculated for each individual study. Early-stage HCC was defined as Barcelona Clinic Liver Cancer (BCLC) stage 0 or A [17], or solitary HCC <5 cm or with up to three nodules <3 cm according to the Milan criteria [18]. The pooled sensitivity and specificity were calculated and the summary receiver operating characteristics curve was acquired using hierarchical models. Study heterogeneity was assessed by Higgins *I*^2^ statistic (*I*^2^ > 50%: substantial heterogeneity). The presence of a threshold effect was evaluated by visual assessment of the coupled forest plots. In addition, we evaluated the presence of threshold effect by the Spearman correlation coefficient between false-positive rate and sensitivity (i.e., 1−specificity). A correlation coefficient >0.6 was considered to represent a considerable threshold effect.

To compare the performance of AMRI according to AMRI protocols (HBP-AMRI vs. NC-AMRI), the HBP-AMRI and NC-AMRI results of all studies were separated and analyzed. The pooled sensitivity and specificity of HBP-AMRI and NC-AMRI were calculated and then compared using joint-model bivariate meta-regression.

When substantial heterogeneity was noted, meta-regression analysis was performed to investigate the causes of study heterogeneity. The meta-regression analysis considered the following 10 covariates: (1) study design (retrospective vs. prospective); (2) study location (Western vs. Eastern countries); (3) study quality (low/unclear risk of bias vs. high risk of bias); (4) cirrhosis (exclusively enrolling patients with cirrhosis vs. others); (5) the most common underlying liver disease (hepatitis B vs. hepatitis C); (6) prevalence of HCC (<20% vs. >20%); (7) MRI magnet field strength (only 1.5T vs. 3.0T or both 1.5 and 3.0T); (8) number of surveillance rounds (single vs. multiple); (9) interpretation of AMRI (clinical practice vs. simulation); and (10) reference standard for HCC (pathology-only vs. pathology or imaging).

Deeks’ funnel plot and Deeks’ asymmetry test were used to evaluate the presence of publication bias. Stata version 16.0 (StataCorp LP, College Station, TX, USA) was used for the statistical analyses.

## 3. Results

### 3.1. Literature Search

**Figure 1:**
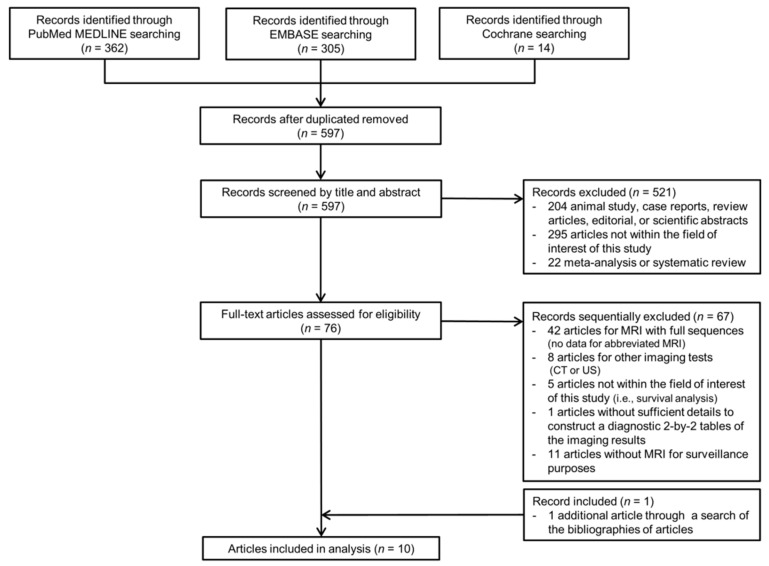
PRISMA diagram of the eligible article selection. CT, computed tomography; US, ultrasonography; MRI, magnetic resonance imaging.

Of the 681 articles identified by the search strategies, 597 articles were found after removing duplicate articles, and 521 articles were further excluded based on titles and abstracts (Figure 1). Sixty-seven articles were excluded during a full-article review. Specifically, studies reporting the performance of AMRI, but those conducted in the retrospective diagnostic cohorts were excluded [19,20,21,22,23,24,25]. In search of the bibliographies, we found one additional eligible article. Finally, a total of 10 eligible articles reported the diagnostic performance of AMRI in HCC surveillance [11,12,13,26,27,28,29,30,31,32].

The study characteristics of 10 studies are shown in Table 1. Among a total of 1547 subjects who underwent surveillance, 213 developed HCC. Two studies were prospective by design [11,31], and five studies exclusively included patients with cirrhosis [13,27,28,30,31]. The most common underlying liver disease was hepatitis C in four studies [12,26,31,32] and hepatitis B in four studies [11,13,29,30]. Three studies used only 1.5-T MRI scanners [13,27,31]. Of the 10 included studies, three used HBP-AMRI protocols [12,26,29] and six used NC-AMRI protocols [11,13,27,28,30,31], with one study using both [32]. Two studies only used pathology as the reference standard for the diagnosis of HCC [27,28], two only used imaging [30,31], and six used both pathological diagnosis and imaging as the reference standard [11,12,13,26,29,32]. Two studies performed multiple surveillance rounds [12,13], with surveillance intervals ranging between 5 and 8.8 months.

### 3.2. Quality Assessment

The results of the study qualities of the 10 included studies are shown in Figure S1. Of the 10 included studies, five had a high risk of bias in at least one of the four domains [12,26,29,30,31]. In the patient-selection domain, three studies had an unclear risk of bias because they were unclear about whether patients were consecutively or randomly enrolled or not [27,28,31]. In the reference standard domain, seven studies were unclear about whether the results of reference standard were determined without knowledge of the index test results [11,12,26,28,30,31,32], and two studies only used multiphase CT or MRI as a reference standard [30,31]. In the flow and timing domain, three studies had a high risk of bias because of an inappropriate time interval between the reference standard and index test (i.e., approximately 1 year), and a failure to use the same reference standard [12,26,29].

### 3.3. Performance of AMRI for Detecting HCC

**Figure 2:**
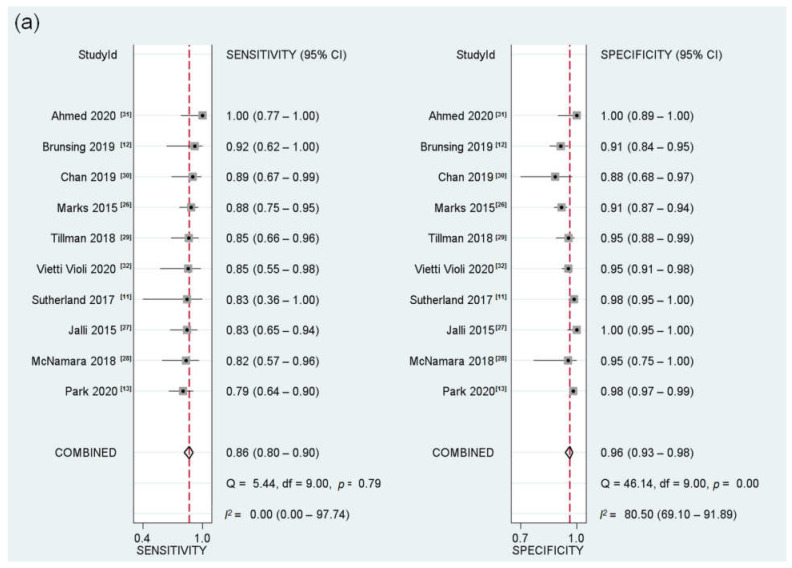

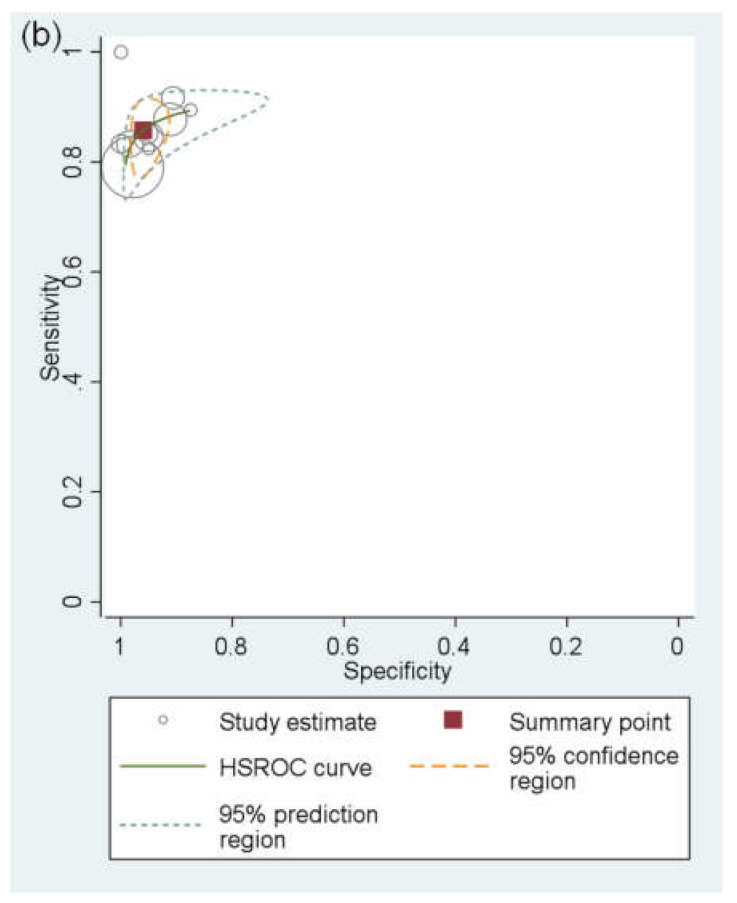
Coupled forest plots and HSROC curve for surveillance AMRI. (**a**) Coupled forest plots of surveillance AMRI for detecting any-stage HCC. (**b**) HSROC curve for the diagnostic accuracy of surveillance AMRI for detecting any-stage HCC. HSROC, Hierarchical Summary Receiver Operating Characteristics; AMRI, abbreviated magnetic resonance imaging; HCC, hepatocellular carcinoma.

**Figure 3:**
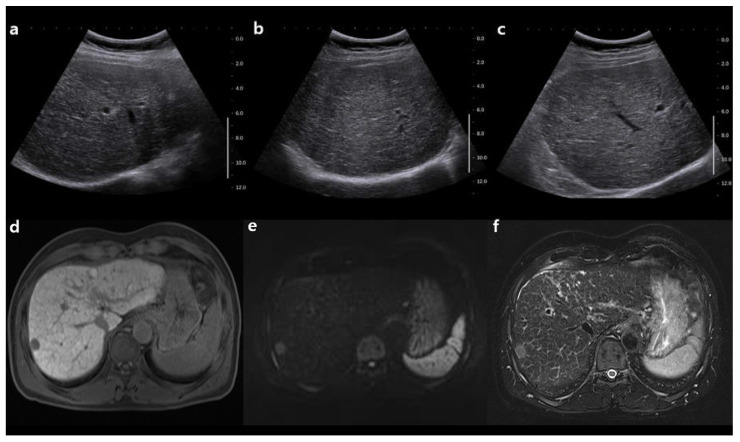
A 64-year-old man with chronic hepatitis B. (**a**–**c**) Surveillance ultrasonography does not show any focal hepatic lesion, whereas (**d**–**f**) surveillance AMRI shows a 1.5 cm nodule in segment VII. Ultrasonography shows coarse echotexture of the liver **(a)**, but it shows no focal hepatic lesion on the right lobe upper view **(b)** and lower view **(c)**. The nodule has hypointensity on the hepatobiliary phase image (**d**), restriction on the diffusion-weighted image (**e**), and moderate hyperintensity on the T2-weighted image (**f**). The surveillance AMRI enabled us to make a diagnosis of HCC.

For all 10 included studies (213 HCCs in 1547 patients) [10,11,12,13,19,20,21,22,23,24], the pooled sensitivity and specificity of AMRI for detecting any-stage HCC were 86% (95% CI, 80–90%; *I*^2^ = 0%) and 96% (95% CI, 93–98%; *I*^2^ = 80.5%), respectively (Figure 2). Four studies (71 HCCs in 752 patients) reported the performance of AMRI for the detection of early-stage HCC [11,12,13,28], showing a pooled sensitivity and specificity of 81% (95% CI, 69–89%; *I*^2^ = 0%) and 97% (95% CI, 93–99; *I*^2^ = 85.5%), respectively (Figure 3). No significant threshold effect was found between sensitivity and specificity (rho = 0.28; *p* = 0.43).

### 3.4. HBP-AMRI vs. NC-AMRI for Detecting HCC

For the detection of any-stage HCC, four studies reported the performance of HBA-AMRI and seven reported the performance of NC-AMRI. Of the four HBP-AMRI studies, three used T2WI, DWI, and HBP [12,26,32], and one used T2WI and HBP [29]. Of the seven NC-AMRI studies, three used T2WI and DWI [13,31,32], two only used DWI [11,28], and two used T2WI, DWI, and T1-weighted dual gradient-echo images [27,30].

The pooled sensitivity and specificity of HBP-AMRI for detecting any-stage HCC were 87% and 93%, respectively, whereas those of NC-AMRI were 82% and 98%, respectively (Table 2). HBP-AMRI had significantly higher sensitivity than NC-AMRI (87% vs. 82%), but significantly lower specificity (93% vs. 98%) (*p* = 0.03). For the detection of early-stage HCC, HBP-AMRI had significantly higher sensitivity than NC-AMRI (87% vs. 79%), but significantly lower specificity (91% vs. 98%) (*p* = 0.02). For the detection of very early-stage HCC, one study reported the performance of HBA-AMRI (sensitivity = 75%, specificity = 91%) and two reported that of NC-AMRI (sensitivity = 59–67%, specificity = 95–98%) (Table S2). Due to a lack of eligible studies, the pooled sensitivity and specificity for detecting very early-stage HCC could not be calculated.

### 3.5. Meta-regression Analysis

The meta-regression analysis results for the diagnostic performance of AMRI are shown in Table 3. Study quality and MRI magnet field strength were significant factors for study heterogeneity (*p* ≤ 0.01). Studies with a low or unclear risk of bias had lower sensitivity (82% vs. 89%) and higher specificity (98% vs. 92%) than those with a high risk of bias. In addition, studies using 1.5T MRI showed lower sensitivity than those using 3.0T or both 1.5T and 3.0T MRI (84% vs. 87%), but a higher specificity (98% vs. 93%). Studies exclusively enrolling patients with cirrhosis showed similar sensitivity to those also enrolling other patients (85% vs. 86%; *p* = 0.34). 

No significant publication bias was found across the studies (*p* = 0.56) (Figure S2).

## 4. Discussion

Our meta-analysis showed that surveillance AMRI had a good overall diagnostic performance for detecting HCC, with pooled sensitivities for detection of any-stage and early-stage HCC of 86% (95% CI, 80–90%) and 81% (95% CI, 69–89%), respectively. Both HBP-AMRI and NC-AMRI protocols demonstrated acceptable diagnostic performance for HCC surveillance, and would therefore be clinically useful for HCC surveillance.

We found that surveillance AMRI showed a high sensitivity for any-stage and early-stage HCC, without statistical heterogeneity across the studies (*I*^2^ for sensitivity = 0%). The results of our analyses can be usefully applied to HCC surveillance in clinical practice because we restricted the scope of our meta-analysis to studies evaluating the performance of MRI for surveillance purposes. In our results, the pooled sensitivity of AMRI for early-stage HCC detection was 81%, which was remarkably higher than that of US reported in a previous meta-analysis (47%) while maintaining high specificity [8]. In addition, the performance of AMRI in our study was similar to that of MRI in a previous prospective study using a complete MRI with full sequences (sensitivity of 81% vs. 85.7%, respectively, and specificity of 97% vs. 97% for early-stage HCC) [9]. Given the advantages of AMRI examinations over full MRI examinations, such as reduced scanner time (i.e., approximately 10 min or less of scan time), reduced cost, less complexity, and simplified workflow (i.e., no need for a power injector for contrast media), AMRI can be considered a cost-effective strategy. Likewise, recent studies suggested that AMRI could be the most cost-effective test for HCC surveillance for high- and intermediate-risk patients with cirrhosis [33], or in a conservative surveillance scenario [34]. Therefore, considering our results together with those of recent cost-effectiveness studies, AMRI may be clinically useful for HCC surveillance, but further prospective studies for evaluating both the diagnostic performance and cost-effectiveness of AMRI in comparison with US in HCC surveillance cohorts are still necessary.

Our results showed that HBP-AMRI demonstrated significantly higher sensitivity than NC-AMRI, at the expense of significantly lower specificity, although both protocols showed acceptable performance for HCC surveillance. The higher sensitivity of HBP-AMRI is largely attributable to the high contrast-to-noise ratio of HBP, which aids in lesion detection. However, because dysplastic nodules and confluent fibrosis can also show HBP hypointensity, HBP-AMRI may result in false-positive diagnoses [32]. In addition, in patients with advanced cirrhosis, who can have reduced hepatocyte function, the hepatocyte uptake of contrast agents is limited, which may hinder the detection of HCC [35]. By comparison, NC-AMRI offers the benefits associated with avoiding the use of a gadolinium-based contrast agent, such as cost-saving and the elimination of the potential risk of long-term retention in human tissues [36], or nephrogenic systemic fibrosis [37]. However, NC-AMRI has a relatively low lesion-to-liver contrast, and some HCCs may be isointense to the liver on T2WI [38] or obscured by heterogeneous background liver parenchymal signal caused by advanced cirrhosis [35], which explains the relatively low sensitivity of NC-AMRI. In addition, DWI, the key sequence in NC-AMRI acquisitions, is vulnerable to artifacts, has blind spots, including the liver dome [39], and early-stage HCC may not exhibit diffusion restriction [40,41]. Taken together, AMRI protocols should be selected with consideration of the advantages and disadvantages of each protocol, and future studies are needed to determine which protocol is better for HCC surveillance.

Meta-regression analysis revealed that study quality as well as MRI magnet field strength were significant factors affecting study heterogeneity. As between-study differences in the use of blinding or in the way the outcomes are defined and measured may lead to differences in the observed measurements, study heterogeneity could be associated with different degrees of bias [42]. Regarding the MRI magnetic field strength, 1.5T MRI has a lower signal-to-noise ratio and lower lesion-to-liver contrast in comparison with 3.0T MRI, which may explain the relatively lower sensitivity of 1.5T MRI compared with 3.0T MRI [43,44].

There are some limitations to our study. First, we could not evaluate the performance of dynamic contrast-enhanced AMRI, which includes pre-contrast, arterial-phase, portal venous-phase, and delayed-phase imaging in HCC surveillance because of a lack of eligible studies, i.e., studies assessing the performance of dynamic contrast-enhanced AMRI acquired for surveillance purposes. Second, the specificity was affected by substantial study heterogeneity; hence, caution was needed when determining the exact pooled specificity of AMRI. To overcome this limitation, we robustly performed further analyses, such as meta-regression. On the contrary, sensitivity was not affected by statistical heterogeneity, and sensitivity is generally considered to be of more importance than specificity in a surveillance setting. Third, although our study evaluated the diagnostic performance of AMRI for detecting HCC, the cost-effectiveness of AMRI should be evaluated before the implementation of AMRI in an HCC surveillance program. Fourth, the comparison between the performance of the HBP-AMRI and NC-AMRI might have been statistically underpowered due to the small number of the included studies and the indirect comparative design.

## 5. Conclusions

In conclusion, surveillance AMRI had a good overall diagnostic performance for detecting both any-stage HCC and early-stage HCC. For detecting HCC, HBP-AMRI had significantly higher sensitivity but lower specificity than NC-AMRI. Therefore, the selection of the AMRI protocol should be determined by considering the advantages of each protocol.

## Figures and Tables

**Table 1:** Study characteristics of the 10 included studies.

Study	Study Design	Study Location (Period)	No. of Patients (% Male)	Cirrhosis Patients (% Cirrhosis)	Most Common Underlying Liver Disease (%)	No. of Patients with HCC (%)	% of HCC < 2 cm	Patient Age, Years *	MRI Magnet	MRI Sequences	AMRI Protocol	Reference Standards for HCC (%)	Interpretation of AMRI
**Marks (2015) [26]**	Retrospective	United States (2008–2012)	298 (56.4)	Cirrhosis (NR) or other risk factors for HCC ^†^	Hepatitis C virus (50.7)	49 (16.4)	28.6	55.9 ± 10.9	1.5 or 3.0-T	T2WI, HBP, DWI	HBP-AMRI	Pathology, multiphase CT, or MRI	Simulation
**Jalli (2015) [27]**	Retrospective	Iran (2011–2013)	96 (NR)	Cirrhosis only (100)	NR	30 (31.3)	NR	NR	1.5-T	T2WI, T1 Dual-GRE, DWI	NC-AMRI	Pathology (100)	Simulation
**Sutherland (2017) [11]**	Prospective	Australia (NR)	192 (72.4)	Cirrhosis (NR) or other risk factors for HCC	Hepatitis B virus (56.3)	6 (3.1)	57.1	58 (22–80), mean (range)	NR	DWI	NC-AMRI	Pathology, multiphase CT, or MRI	Clinical practice
**McNamara (2018) [28]**	Retrospective	United States (2009–2013)	37 (67.6)	Cirrhosis only (100)	NR	17 (45.9)	NR	21–70, range	1.5 or 3.0-T	DWI	NC-AMRI	Pathology (100)	Simulation
**Tillman (2018) [29]**	Retrospective	United States (2008–2014)	79 (53.2)	Cirrhosis (64.6) or other risk factors for HCC	Hepatitis B virus (41.8)	13 (16.5)	44.4	57.5 ± 13.7	1.5 or 3.0-T	T2WI, HBP	HBP-AMRI	Pathology (59.3), multiphase CT, or MRI (40.7)	Simulation
**Brunsing (2019) [12]**	Retrospective	United States (2014–2016)	141 (54.6)	Cirrhosis (92.9) or other risk factors for HCC	Hepatitis C virus (37.9)	12 (8.5)	66.7	59.1 ± 11.5	1.5 or 3.0-T	T2WI, HBP, DWI	HBP-AMRI	Pathology, multiphase CT, or MRI	Clinical practice
**Chan (2019) [30]**	Retrospective	Australia (2015–2018)	44 (49.5)	Cirrhosis only (100)	Hepatitis B virus (14.9)	20 (45.5)	40.5	63 ± 13	3.0-T	T2WI, DWI, T1 Dual-GRE	NC-AMRI	Multiphase MRI (100)	Simulation
**Ahmed (2020) [31]**	Prospective	Egypt (2018–2019)	41 (53.7)	Cirrhosis only (100)	Hepatitis C virus (100)	10 (24.4)	NR	53.4 ± 9.2	1.5-T	T2WI, DWI	NC-AMRI	Multiphase MRI (100)	NR
**Park (2020) [13]**	Retrospective	Korea (2011–2014)	382 (56.8)	Cirrhosis only (100)	Hepatitis B virus (72.3)	43 (11.3)	83.3	56.4, median	1.5-T	T2WI, DWI	NC-AMRI	Pathology (46.5) or multiphase CT (53.5)	Simulation
**Vietti Violi (2020) [32]**	Retrospective	United States (2017)	237 (58.6)	Cirrhosis (87.3) or other risk factors for HCC	Hepatitis C virus (25.7)	13 (5.5)	NR	58 ± 11.9	1.5 or 3.0-T	T2WI, HBP, DWI	HBP-AMRI and NC-AMRI	Pathology (7.7), multiphase CT, or MRI (92.3)	Simulation

* Unless otherwise indicated, data are mean ± standard deviation. ^†^ Other risk factors for HCC included chronic hepatitis B or C, alcoholic liver disease, and non-alcoholic steatohepatitis. HCC, hepatocellular carcinoma; MRI, magnetic resonance imaging; AMRI, abbreviated MRI; T2WI, T2-weighted imaging; HBP, hepatobiliary phase; DWI, diffusion-weighted imaging; HBP, hepatobiliary phase; CT, computed tomography; NR, not reported; T1 Dual-GRE, T1-weighted dual gradient-echo in-phase and out-of-phase imaging; NC, non-contrast.

**Table 2:** Diagnostic performance of AMRI for detecting hepatocellular carcinoma.

**Any Stage HCC**					
**HBP AMRI**			**NC AMRI**		
**Study**	**Sensitivity (95% CI)**	**Specificity (95% CI)**	**First author**	**Sensitivity (95% CI)**	**Specificity (95% CI)**
Marks [26]	88% (75,95)	91% (87,94)	Jalli [27]	83% (65,94)	100% (95,100)
Tillman [29]	85% (66,96)	95% (88,99)	Sutherland [11]	83% (36,100)	98% (95,100)
Brunsing [12]	92% (62,100)	91% (84,95)	McNamara [28]	82% (57,96)	95% (75,100)
Vietti Violi [32] *	85% (55,98)	95% (91,98)	Chan [30]	89% (67,99)	88% (68,97)
			Ahmed [31]	100% (77,100)	100% (89,100)
			Park [13]	79% (64,90)	98% (97,99)
			Vietti Violi [32] *	62% (32,86)	96% (92,98)
Pooled estimates	87% (81,94)	93% (91,95)		82% (76,89)	98% (96,99)
**Early-stage HCC**					
**HBP AMRI**			**NC AMRI**		
**Study**	**Sensitivity (95% CI)**	**Specificity (95% CI)**	**First author**	**Sensitivity (95% CI)**	**Specificity (95% CI)**
Brunsing [12]	87% (65,100)	91% (86,96)	Sutherland [11]	80% (28,99)	98% (95,100)
			McNamara [28]	81% (54,96)	95% (76,100)
			Park [13]	79% (63,90)	98% (97,99)
Pooled estimates	87% (65,100)	91% (86,96)		79% (69,89)	98% (97,99)

* In the study by Vietti Violi et al. [32], the diagnostic accuracies of both HBP and NC-AMRI were reported separately. AMRI, abbreviated magnetic resonance imaging; HCC, hepatocellular carcinoma; HBP, hepatobiliary phase; NC, non-contrast; CI, confidence interval.

**Table 3:** Meta-regression analysis of abbreviated MRI for detecting hepatocellular carcinoma.

Summary Estimate				
Variables	Subgroup	Sensitivity (95% CI)	Specificity (95% CI)	*p*-Value
Study design	Prospective (*n* = 2)	95% (86,100)	99% (97,100)	0.05
	Retrospective (*n* = 8)	85% (80,90)	95% (93,97)	-
Study location *	Western (*n* = 7)	87% (81,92)	94% (92,97)	0.06
	Eastern (*n* = 2)	81% (72,90)	98% (97,100)	-
Study quality	Low/unclear risk of bias (*n* = 5)	82% (74,89)	98% (97,98)	0.01
	High risk of bias (*n* = 5)	89% (84,95)	92% (90,94)	-
Cirrhosis	Exclusively enrolling cirrhosis patients (*n* = 5)	85% (78,92)	98% (96,99)	0.34
	Others ^†^ (*n* = 5)	86% (79,93)	95% (92,97)	-
Most common underlying liver disease *	Hepatitis C (*n* = 4) Hepatitis B (*n* = 4)	90% (83,96) 83% (76,91)	93% (91,96) 97% (96,99)	0.13
HCC prevalence in each study	<20% (*n* = 6) >20% (*n* = 4)	85% (78,91) 88% (80,96)	96% (93,98) 97% (94,100)	0.53
MRI magnet field strength *	Only 1.5T (*n* = 3)	84% (76,92)	98% (97,99)	<0.01
	3.0T or both 1.5 and 3.0T (*n* = 6)	87% (81,93)	93% (91,95)	-
Number of surveillance rounds	Single (*n* = 8)	87% (81,92)	96% (94,99)	0.80
	Multiple (*n* = 2)	83% (72,94)	96% (92,100)	-
Interpretation of AMRI *	Clinical practice (*n* = 2)	88% (73,100)	96% (91,100)	0.91
	Simulation (*n* = 7)	85% (79,90)	96% (93,98)	-
Reference standard for HCC	Pathology-only (*n* = 2)	83% (72,94)	99% (96,100)	0.36
	Pathology or imaging (*n* = 8)	86% (81,92)	95% (93,98)	-

* Studies not reporting relevant data were excluded. ^†^ Studies that included patients at-risk other than those with cirrhosis. AMRI, abbreviated magnetic resonance imaging; CI, confidence interval; HCC, hepatocellular carcinoma.

## Data Availability

All data accessed are available in the article and its Supplementary Materials.

## Supplementary Materials

The following are available online at https://www.mdpi.com/article/10.3390/cancers13122975/s1. Figure S1: Results of quality assessments of the articles according to QUADAS-2 criteria, Figure S2: Deeks’ funnel plot to evaluate the publication bias of surveillance abbreviated magnetic resonance imaging, Table S1: Search queries, Table S2: Diagnostic performance of abbreviated magnetic resonance imaging for detecting very early-stage hepatocellular carcinoma.
