# Supplementary material for: Meta-Analysis of the Accuracy of Abbreviated Magnetic Resonance Imaging for Hepatocellular Carcinoma Surveillance: Non-Contrast versus Hepatobiliary Phase-Abbreviated Magnetic Resonance Imaging
Source: Cancers (Basel). 2021 Jun 14;13(12):2975. doi: 10.3390/cancers13122975 (PMC8231787; doi:10.3390/cancers13122975)
Supplement: Supplementary file 1 [file cancers-13-02975-s001.zip › cancers-1226151-suppl. conversion.pdf]

Supplementary Materials: Meta-Analysis of the Accuracy of Abbreviated Magnetic Resonance Imaging for Hepatocellular Carcinoma Surveillance: Non-Contrast Versus Hepatobiliary Phase-Abbreviated Magnetic Resonance Imaging

Dong Hwan Kim, Sang Hyun Choi, Ju Hyun Shim, So Yeon Kim, Seung Soo Lee, Jae Ho Byun and Joon-Il Choi

Table S1. Search queries.

No.	Search queries for MEDLINE
#1	"Carcinoma, Hepatocellular"[Mesh] OR "Liver Neoplasms"[Mesh:NoExp]
#2	(liver*[TW] OR hepatic*[TW] OR Hepato*[TW]) AND (carcinoma*[TW] OR cancer[TW] OR cancers[TW] OR tumor[TW] OR neoplas*[TW] OR malignan*[TW])
#3	hepatocarcinoma*[TW] OR hepatoma*[TW] OR "liver carcinoma"[TW] OR HCC[TW]
#4	#1 OR #2 OR #3
#5	"Magnetic Resonance Imaging"[Mesh]
#6	Magnetic-Resonanc*[TW] OR "MR"[TI] OR "MRI"[TW] OR "diffusion-weighted"[TW]
#7	#5 OR #6
#8	abbreviat*[TW] OR surveillanc*[TW] OR screen*[TW]
#9	"Early Detection of Cancer"[Mesh] OR "mass screening"[MeSH] OR "Epidemiological Monitoring"[Mesh]
#10	#8 OR #9
#11	"Predictive Value of Tests"[Mesh] OR "Sensitivity and Specificity"[Mesh]
#12	predictive value*[TW] OR detection rate*[TW] OR "False Negative"[TW] OR "False positive"[TW] OR "True Negative"[TW] OR "True positive"[TW] OR "PPV"[TW] OR "NPV"[TW] OR Sensitivit*[TW] OR Specificit*[TW]
#13	"Reproducibility of Results"[Mesh]
#14	accurac*[TW] OR Validit*[TW]
#15	#11 OR #12 OR #13 OR #14
#16	#4 AND #7 AND #10 AND #15
#17	#16 AND ("2000/01/01"[PDAT] : "3000/12/31"[PDAT]) AND (English[Lang])
No.	Search queries for EMBASE
#1	'liver cell carcinoma'/exp OR 'liver cancer'/de
#2	((liver* OR hepatic* OR Hepato*) NEAR/6 (Carcinoma* OR Cancer* OR tumor* OR Neoplas* OR malignan*)):ab,ti,kw
#3	(hepatocarcinoma* OR hepatoma* OR 'liver carcinoma' OR HCC):ab,ti,kw
#4	#1 OR #2 OR #3
#5	'nuclear magnetic resonance imaging'/exp OR 'MR':ti OR 'MRI':ab,ti,kw
#6	(Magnetic-Resonanc* OR 'diffusion-weighted'):ab,ti,kw
#7	#5 OR #6
#8	(abbreviat* OR surveillanc* OR screen*):ab,ti,kw
#9	'early cancer diagnosis'/exp OR 'cancer screening'/exp OR 'disease surveillance'/exp
#10	#8 OR #9
#11	'diagnostic accuracy'/exp OR 'predictive value'/exp OR 'sensitivity and specificity'/de
#12	('predictive value' OR 'predictive values' OR 'detection rate' OR 'detection rates' OR 'False Negative' OR 'False positive' OR 'True Negative' OR 'True positive' OR 'PPV' OR 'NPV' OR Sensitivit* OR Specificit*):ab,ti,kw
#13	(Diagnos* NEAR/3 Accurac*):ab,ti,kw
#14	(Validit*):ab,ti,kw
#15	#11 OR #12 OR #13 OR #14
#16	#4 AND #7 AND #10 AND #15
#17	#16 AND ([english]/lim) AND [2000-2020]/py AND ([article]/lim OR [article in press]/lim OR [review]/lim)
No.	Search queries for Cochrane
#1	[mh "Carcinoma, Hepatocellular"] or [mh ^"Liver Neoplasms"]
#2	((Hepatocellular* or liver-cell* or hepatic-cell* or Hepato-cell*) near/6 (Cancer* or tumor* or Neoplas* or carcinoma*)):ab,ti,kw
#3	hepatocarcinoma*:ab,ti,kw or hepatoma*:ab,ti,kw or "liver carcinoma":ab,ti,kw or HCC:ab,ti,kw
#4	#1 or #2 or #3
#5	[mh "Magnetic Resonance Imaging"]
#6	Magnetic-Resonanc*:ab,ti,kw or "MR":ti or "MRI":ab,ti or "diffusion-weighted":ab,ti,kw
#7	#5 or #6
#8	abbreviat*:ab,ti,kw or surveillanc*:ab,ti,kw or Monitoring*:ab,ti,kw
#9	[mh "Early Detection of Cancer"] or [mh "mass screening"] or [mh "Epidemiological Monitoring"]
#10	#8 or #9
#11	[mh "Predictive Value of Tests"] or [mh "Sensitivity and Specificity"]

#12	(predictive value* or detection rate* or "False Negative" or "False positive" or "True Negative" or "True positive" or "PPV" or "NPV" or Sensitivit* or Specificit*):ab,ti,kw
#13	[mh "Reproducibility of Results"]
#14	(Reproducib* or accurac* or Reliabilit* or Validit*):ab,ti,kw
#15	#11 or #12 or #13 or #14
#16	#4 and #7 and #10 and #15
#17	#16 in Trials(Published); 2000-2020

Table S2. Diagnostic performance of abbreviated magnetic resonance imaging for detecting very early-stage hepatocellular carcinoma.

HBP AMRI			NC AMRI		
**First author**	**Sensitivity [95% CI]**	**Specificity [95% CI]**	**First author**	**Sensitivity [95% CI]**	**Specificity [95% CI]**
Brunsing [1]	75% [19,99]	91% [85,95]	Sutherland [2]	67% [9,99]	98% [95,100]
			Chan [3]	59% [33,82]	95% [90,98]

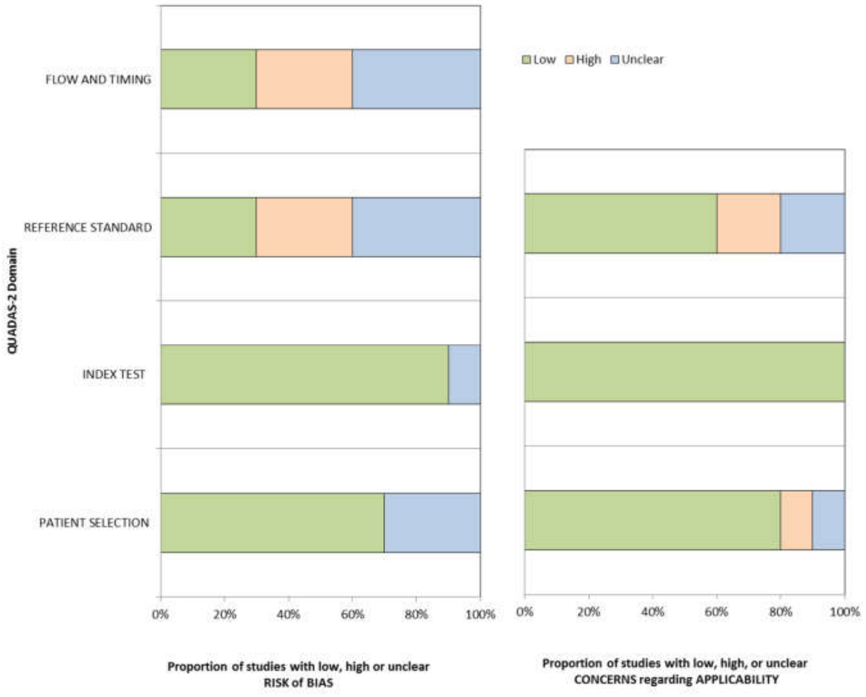

Figure S1. Results of quality assessments of the articles according to QUADAS-2 criteria. The methodological quality of Table 0. with low (i.e., high quality), high, or unclear risk of bias and the proportion of articles with low (i.e., high quality), high, or unclear concerns regarding applicability for each domain.

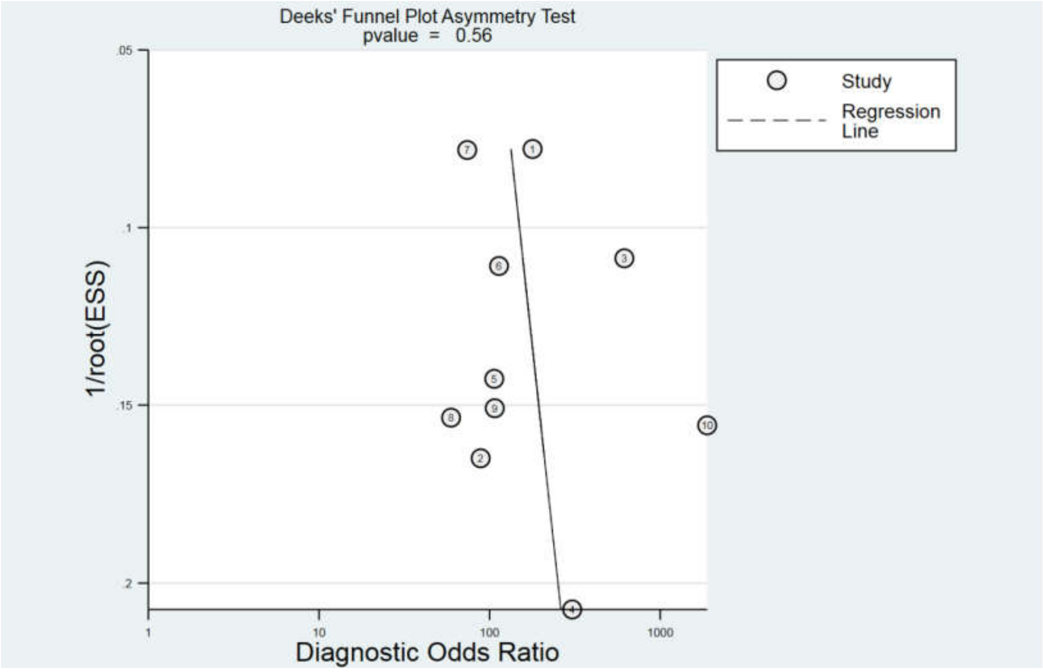

Figure S2. Deeks' funnel plot to evaluate the publication bias of surveillance abbreviated magnetic resonance imaging.

Reference

1. Brunsing, R.L.; Chen, D.H.; Schlein, A.; Wolfson, T.; Gamst, A.; Mamidipalli, A.; Violi, N.V.; Marks, R.M.; Taouli, B.; Loomba, R.; et al. Gadoxetate-enhanced Abbreviated MRI for Hepatocellular Carcinoma Surveillance: Preliminary Experience. *Radiol. Imaging Cancer* **2019**, *1*, e190010, doi:10.1148/rycan.2019190010.
2. Sutherland, T.; Watts, J.; Ryan, M.; Galvin, A.; Temple, F.; Vuong, J.; Little, A.F. Diffusion-weighted MRI for hepatocellular carcinoma screening in chronic liver disease: Direct comparison with ultrasound screening. *J. Med Imaging Radiat. Oncol.* **2017**, *61*, 34–39, doi:10.1111/1754-9485.12513.
3. Chan, M.V.; McDonald, S.J.; Ong, Y.-Y.; Mastrocostas, K.; Ho, E.; Huo, Y.R.; Santhakumar, C.; Lee, A.U.; Yang, J. HCC screening: assessment of an abbreviated non-contrast MRI protocol. *Eur. Radiol. Exp.* **2019**, *3*, 1–11, doi:10.1186/s41747-019-0126-1.
